# Supplementary material for: High-performance ZrNiSn-based half-Heusler thermoelectrics with hierarchical architectures enabled by reactive sintering
Source: Nat Commun. 2025 Jul 15;16:6497. doi: 10.1038/s41467-025-61868-x (PMC12259942; doi:10.1038/s41467-025-61868-x)
Supplement: Supplementary file 1 — Supplementary information [file 41467_2025_61868_MOESM1_ESM.pdf]

Supplementary information

High-Performance ZrNiSn-based Half-Heusler Thermoelectrics with Hierarchical Architectures Enabled by Reactive Sintering

Xin Ai, Yu Wu, Haiyan Lyu, Lars Giebeler, Wenhua Xue, Andrei Sotnikov, Yumei Wang, Qihao Zhang, Denys Makarov, Yuan Yu, G. Jeffrey Snyder, Kornelius Nielsch, Ran He

Single parabolic band model: The single band model was adopted to calculate the relationship between thermoelectric parameters and carrier concentrations (n) at 300 K. The relationship between Seebeck coefficient (α) and carrier concentration (n) is given by[1]

$$\alpha = \pm\frac{k_\mathrm{B}}{e}\left[\frac{\left(\frac{5}{2}+\lambda\right)F_{\frac{3}{2}+\lambda}(\eta)}{\left(\frac{3}{2}+\lambda\right)F_{\frac{1}{2}+\lambda}(\eta)}-\eta\right] \quad (S1)$$

$$n = 4\pi\left(\frac{2m^*k_\mathrm{B}T}{h^2}\right)^{\frac{3}{2}}F_{\frac{1}{2}}(\eta) \quad (S2)$$

$$F_j(\eta) = \int_0^\infty \frac{x^\mathrm{j}}{1+\exp(x-\eta)}\mathrm{d}x \quad (S3)$$

where η is the simple Fermi level, k_B is the Boltzmann constant, m^* is the effective mass, h is the Planck constant, T is the absolute temperature, and λ is the scattering parameter. When the relaxation time approximation is used and the relaxation time and carrier energy satisfy $\tau = \tau_0 E^\lambda$. The alloy scattering gives the value $\lambda = -1/2$.

The Lorenz number *L* is calculated by formula S5[2]:

$$L = [1.5+\exp(-\frac{|\alpha|}{116})]\times 10^{-8} \quad (S5)$$

The sound velocity(v_s) is a weighted average of the longitudinal (v_l) and transverse (v_t) sound velocities, as follows[3]

$$\frac{1}{v_\mathrm{s}} = \left(\frac{1}{v_\mathrm{l}^3+v_\mathrm{t}^3}\right)^{\frac{1}{3}} \quad (S6)$$

Debye temperature (θ_D) is estimated from the average sound velocity v_s by

$$\theta_{\mathrm{D}} = \frac{v_{\mathrm{s}} h}{k_{\mathrm{B}}} \left(\frac{3N}{4\pi V} \right)^{\frac{1}{3}} \tag{S7}$$

Debye model, lattice thermal conductivity can be expressed as:[4]

$$\kappa_{\mathrm{L}} = \frac{k_{\mathrm{B}}}{2\pi^2 v_{\mathrm{s}}} \left(\frac{k_{\mathrm{B}} T}{\hbar} \right)^3 \int_0^{\frac{\theta_{\mathrm{D}}}{T}} \frac{x^4 \mathrm{e}^x}{\tau_{\mathrm{t}}^{-1} (\mathrm{e}^x - 1)^2} \mathrm{d}x \tag{S8}$$

where $x = \hbar\omega/(k_B T)$ is the reduced phonon frequency, ω is phonon frequency, $\hbar$ is reduced Planck constant, θ_D is Debye temperature, v_s is sound velocity, and τ_t is the phonon-scattering relaxation time. Thus, the lattice thermal conductivity is influenced by the phonon-scattering relaxation time from various scattering mechanism. The relationship between different relaxation time can be expressed as,[5,6]

$$\tau_{\mathrm{t}}^{-1} = \tau_{\mathrm{B}}^{-1} + \tau_{\mathrm{P}}^{-1} + \tau_{\mathrm{U}}^{-1} + \tau_{\mathrm{NP}}^{-1} \tag{S9}$$

where τ_{B}, τ_{P}, τ_{U}, and τ_{NP} represent the phonon-scattering relaxation time for grain boundary scattering, point defect scattering, phonon-phonon Umklapp scattering, and nanoprecipitates, respectively.

Grain boundaries scattering can be expressed as:[7]

$$\tau_{\mathrm{B}}^{-1} = \frac{v_{\mathrm{s}}}{d} \tag{S10}$$

d is grain size.

Point defect scattering can be expressed as:[8]

$$\tau_{\mathrm{P}}^{-1} = \frac{AV}{4\pi v_{\mathrm{s}}^3} \omega^4 \tag{S11}$$

where A is the fitting number, V is the average atomic volume.

Phonon-phonon Umklapp scattering can be expressed by following formula,

$$\tau_{\mathrm{U}}^{-1} = \mathrm{B} \frac{\hbar \gamma^2}{M v_{\mathrm{s}}^2 \theta_{\mathrm{D}}} \omega^2 T \exp\left(-\frac{\theta_{\mathrm{D}}}{3T} \right) \tag{S12}$$

where B is the fitting number, γ is the Grüneisen parameter, which characterizes the strength of lattice anharmonic vibrations in materials.

The nanoprecipitates can scatter short and medium wavelength phonons (up to approximately 100 nm). The relaxation time of phonon scattering due to nanoscale precipitates can be

described by a Matthiessen-type combination of short-wavelength scattering and long-wavelength scattering mechanisms:[9]

$$\tau_{\mathrm{NP}}^{-1} = v_{\mathrm{s}}\left(\sigma_{\mathrm{s}}^{-1} + \sigma_{\mathrm{l}}^{-1}\right)^{-1} V_{\mathrm{p}} \quad \text{(S13)}$$

$$\sigma_{\mathrm{s}} = 2\pi R^2 \quad \text{(S14)}$$

$$\sigma_{\mathrm{l}} = \pi R^2 \frac{4}{9} (\frac{\Delta\rho}{\rho})^2 \left(\frac{\omega R}{v_{\mathrm{s}}}\right)^4 \quad \text{(S15)}$$

where C is the fitting number (dimensionless), R is the particles' average radius, ρ is matrix density, $\Delta\rho$ is the density difference between the particle and matrix materials, and V_{p} is the number density of the nanoscale particles. Table S3 lists the fitting parameters.

The theoretical minimum thermal conductivity (κ_{min}) is estimated using the Cahill model:[10]

$$\kappa_{\mathrm{min}} = \left(\frac{\pi}{6}\right)^{\frac{1}{3}} k_B N^{\frac{2}{3}} \sum_i v_i \left(\frac{T}{\theta_i}\right)^2 \int_0^{\frac{\theta_i}{T}} \frac{x^3 e^x}{(e^x - 1)^2} \mathrm{d}x \quad \text{(S16)}$$

In this formula, v_i, θi, and N are the acoustic phonon velocity, the Debye temperature, and the number density of atoms, respectively. Parameter θ_i can be obtained by the relation $\theta_i = v_i(\hbar/k_{\mathrm{B}})(6\pi^2 n)^{\frac{1}{3}}$. n is the number density of atoms. The parameters used for calculating the κ_{min} of HfNiSn are shown in Table S4.

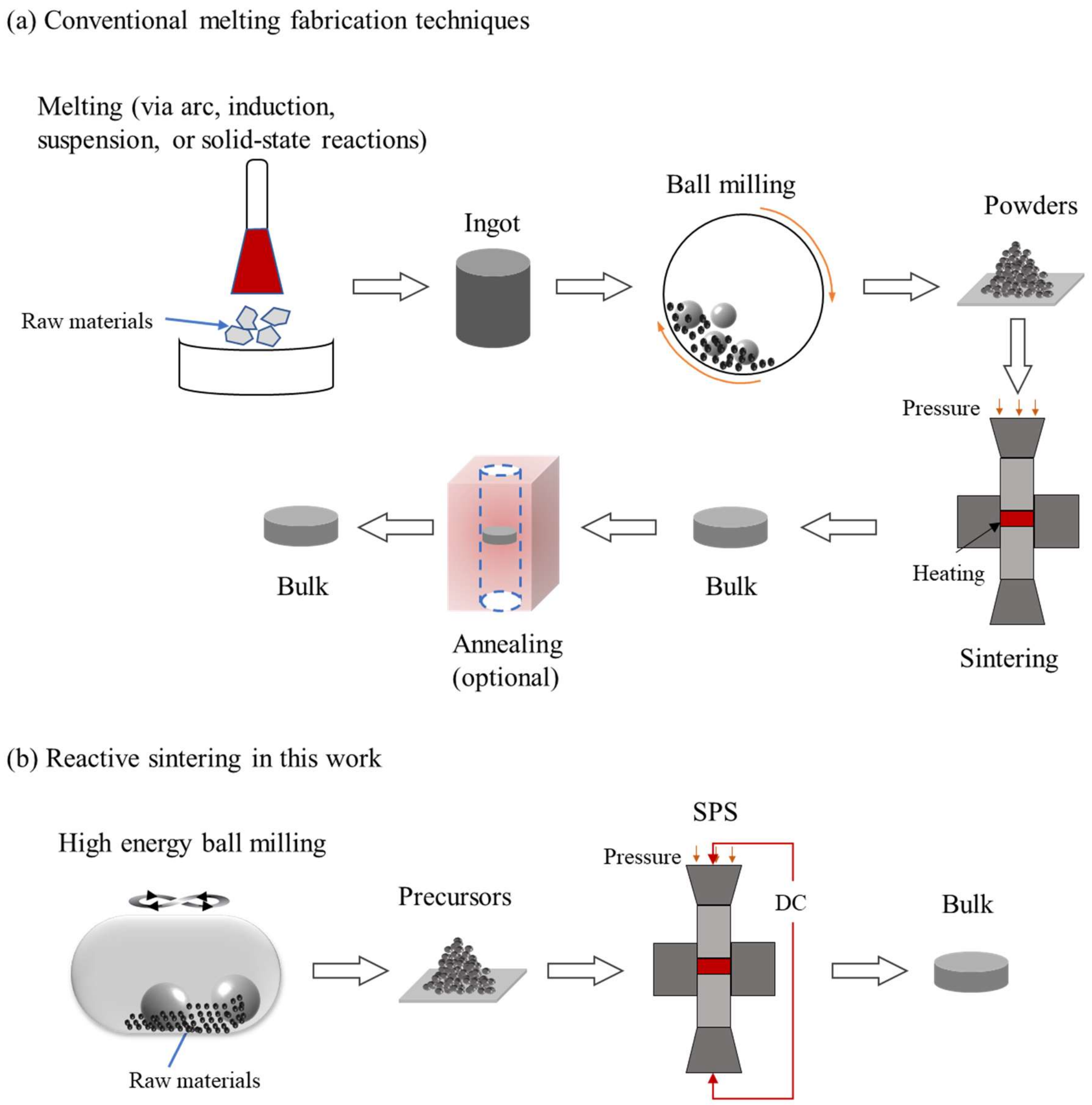

Figure S1. Comparation of fabrication methods for half-Heusler materials. (a) Schematic diagram of conventional melting process for half-Heusler compounds. **(b)** Schematic diagram of short-term ball milling (6 hours) and reactive sintering used in this work.

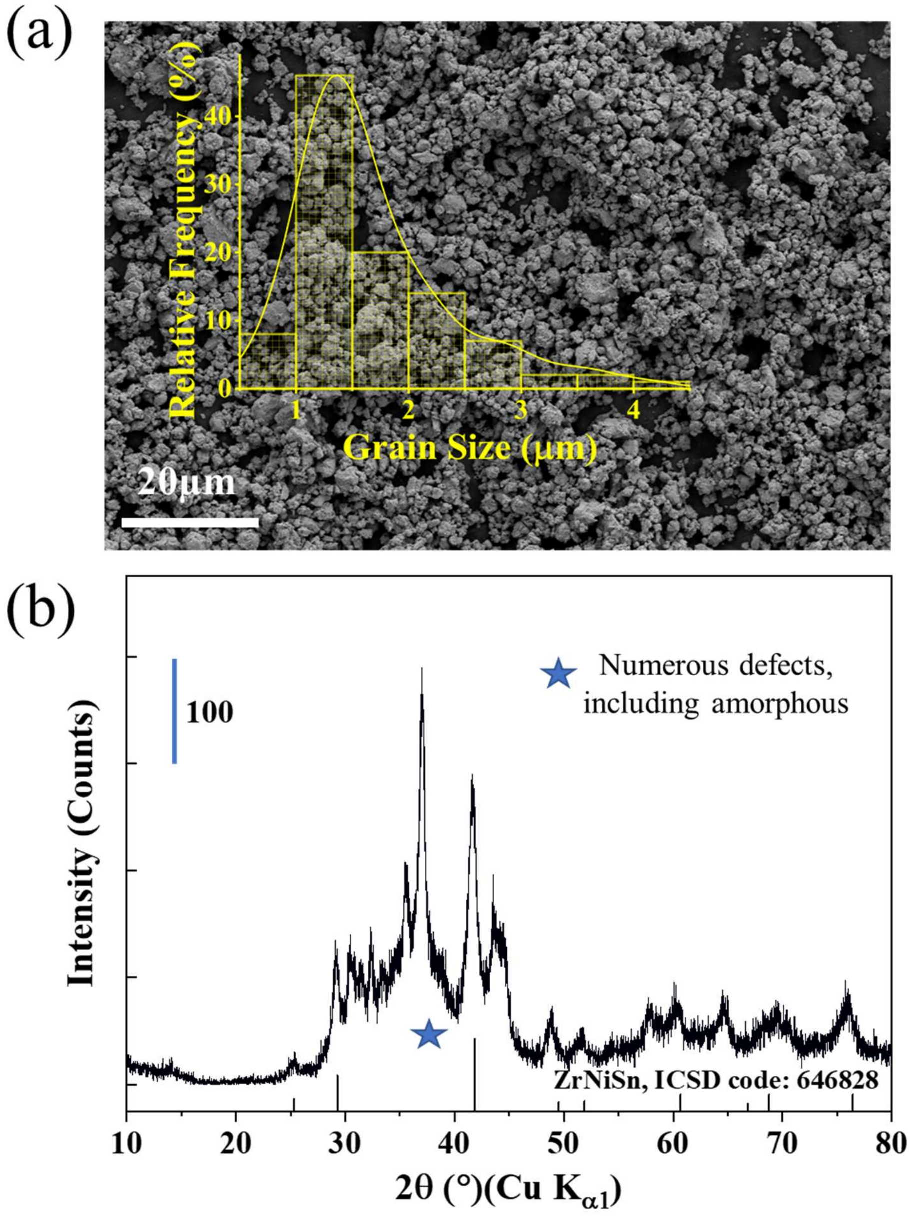

Figure S2. $Zr_{0.75}Hf_{0.25}NiSn_{0.99}Sb_{0.01}$ precursors prepared by mechanical alloying of raw metal powders for 6 hours: **(a)** SEM image and particle size distributions; **(b)** Corresponding XRD pattern.

Table S1. Summary of the density, relative density, longitudinal and transverse sound velocities (v_l, v_t), sound velocity (v_s), Hall carrier concentration (n_H) and Hall mobility (μ_H) of $Zr_{0.75}Hf_{0.25}NiSn_{0.99}Sb_{0.01}$ bulk samples synthesized using different sintering parameters.

Label Name	Sinter Temp. (°C)	Holding Time (minutes)	Density (g/cm^3)	Relative Density	v_l (m/s)	v_t (m/s)	v_s (m/s)	n_H (10^{20} cm^{-3})	μ_H (cm^2 V^{-1} s^{-1})
750 °C	750	12	8.48	99.5%	/	/	/	/	/
800 °C	800	12	8.46	99.3%	5244.8	3018.5	3352.0	3.46	28.83
850 °C	850	12	8.48	99.5%	/	/	/	3.25	29.34
900 °C	900	12	8.49	99.6%	/	/	/	3.43	27.48
950 °C/D100	950	12	8.50	99.8%	5283.4	3043.2	3379.2	3.31	27.93
1000 °C	1000	12	8.51	99.9%	/	/	/	3.07	26.29
1050 °C	1050	12	8.50	99.8%	5298.4	3038.7	3375.5	3.09	25.60
1100 °C	1100	12	8.50	99.8%	/	/	/	/	/
D98	950	6	8.35	98.2%	5098.2	2961.600	3286.1	3.06	29.97
D95	950	4	8.12	95.5%	4691.9	2811.800	3110.9	2.87	28.44
D91	950	2	7.73	90.9%	4730.2	2829.100	3130.6	2.90	27.32

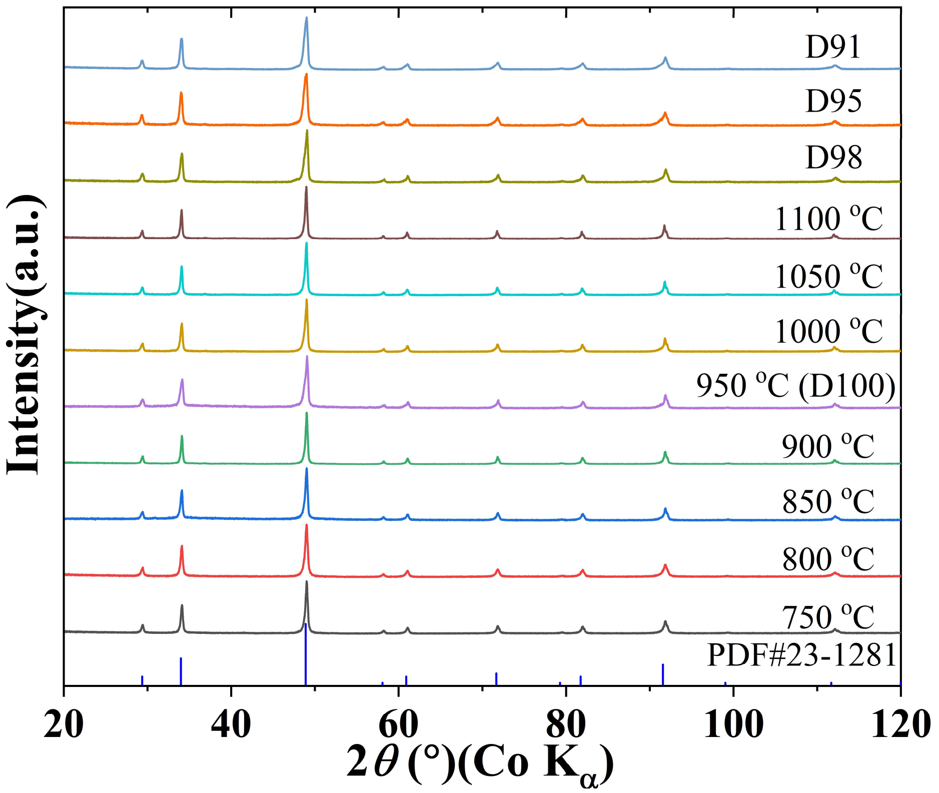

Figure S3. XRD patterns of bulk $Zr_{0.75}Hf_{0.25}NiSn_{0.99}Sb_{0.01}$ samples: 800 °C, 850 °C, 900 °C, 950 °C (D100), 1000 °C, 1050 °C, D98, D95, and D91. The standard ZrNiSn powder diffraction file from Ref. [11] is included.

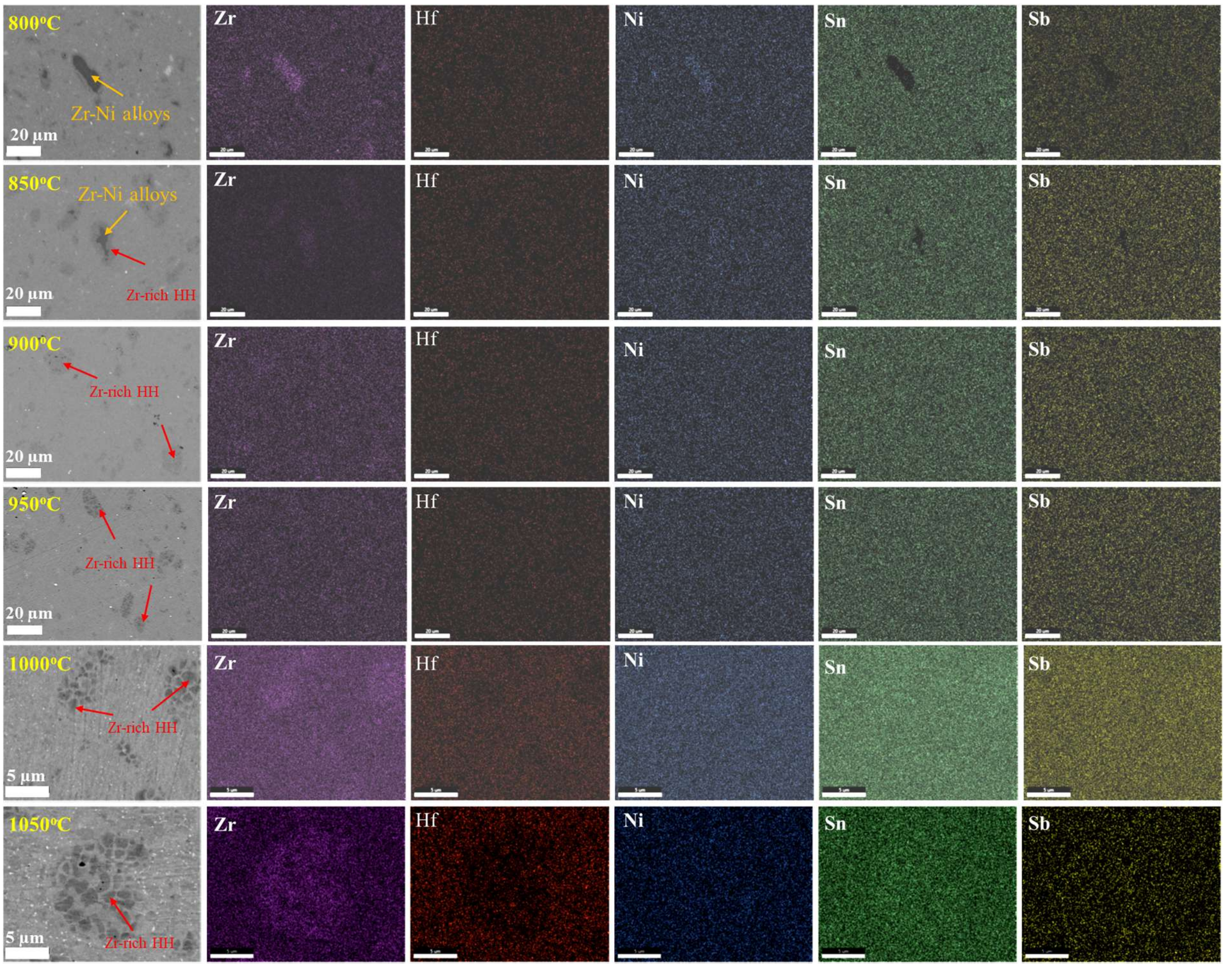

Figure S4. Backscattered electron (BSD) images for $Zr_{0.75}Hf_{0.25}NiSn_{0.99}Sb_{0.01}$ bulk samples sintered at 800 °C, 850 °C, 900 °C, 950 °C, 1000 °C, and 1050 °C, and corresponding elemental mapping results obtained from energy-dispersive X-ray (EDX) analysis.

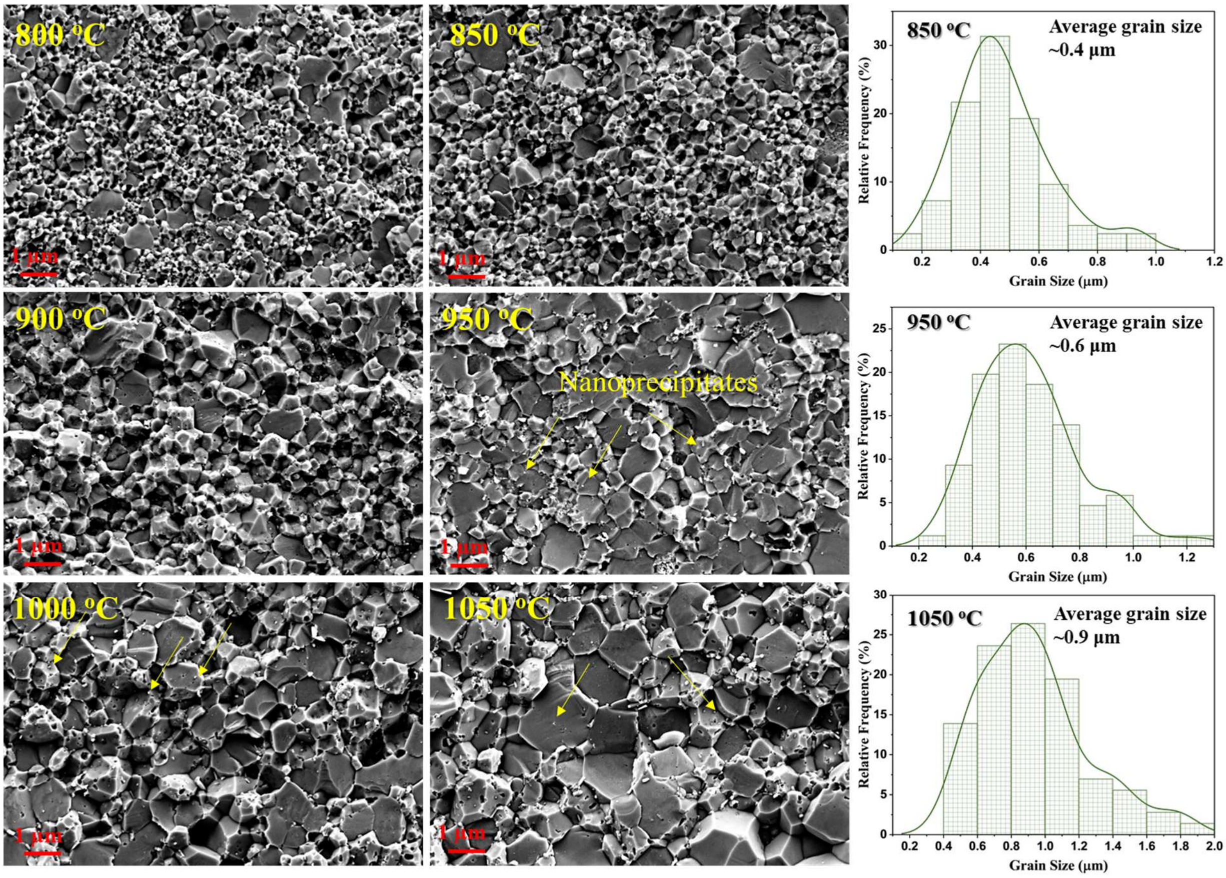

Figure S5. SEM images of fractured surfaces for $Zr_{0.75}Hf_{0.25}NiSn_{0.99}Sb_{0.01}$ bulk samples sintered at 800 °C, 850 °C, 900 °C, 950 °C, 1000 °C and 1050 °C, along with corresponding grain size distribution.

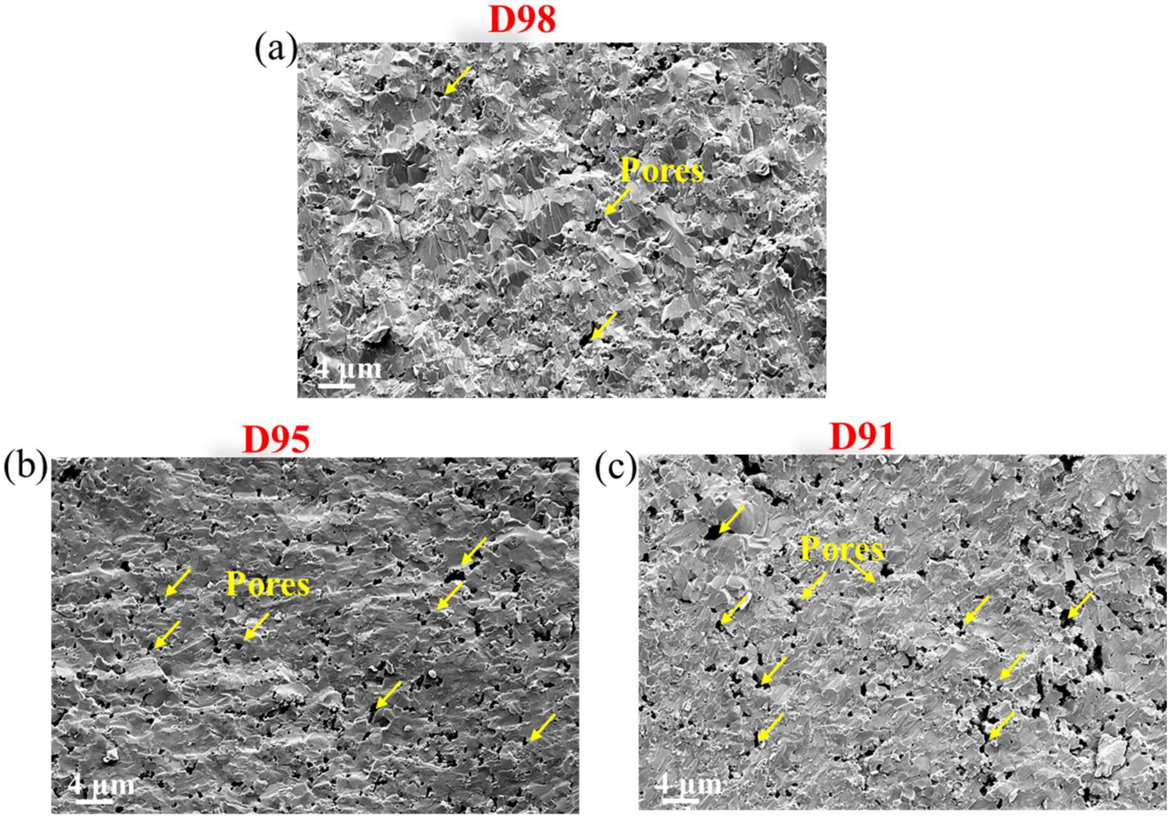

Figure S6. SEM images of fractured surfaces for $Zr_{0.75}Hf_{0.25}NiSn_{0.99}Sb_{0.01}$ bulk samples: (**a**) D98, (**b**) D95, and (**c**) D91.

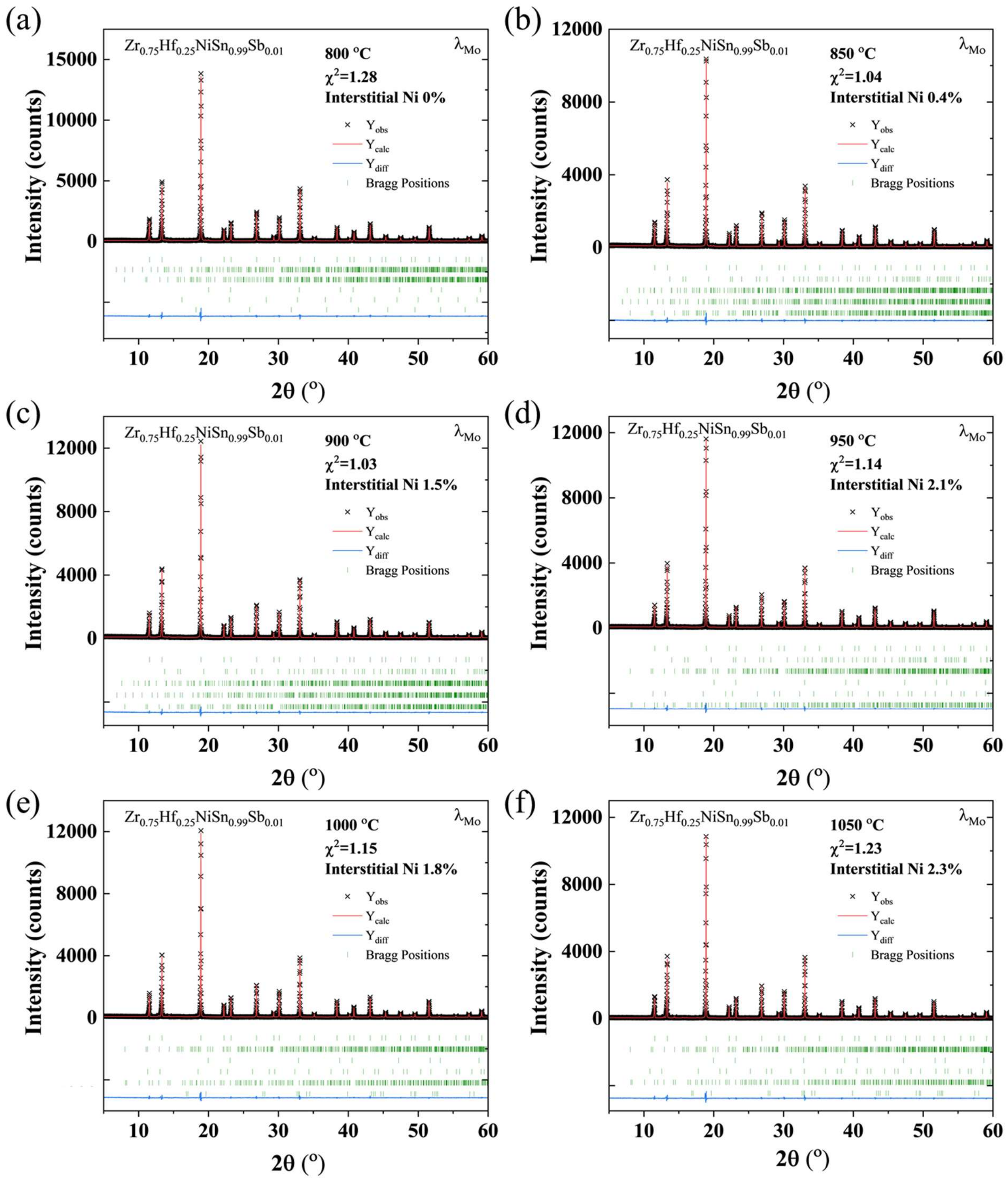

Figure S7. X-ray powder diffraction pattern and results of Rietveld analyses for $Zr_{0.75}Hf_{0.25}NiSn_{0.99}Sb_{0.01}$ samples sintered at different temperatures with a holding time of 12 minutes, with Bragg position markers corresponding to the main half-Heusler and the minor phases. **(a)** 800°C sample with the markers assigned to $Zr_{0.75}Hf_{0.25}NiSn_{0.99}Sb_{0.01}$, HfO_2, $Ni_{3.08}Sn_4$, Ni, Zr, and Sn (from top to bottom). **(b)** 850°C sample with the markers assigned to $Zr_{0.75}Hf_{0.25}NiSn_{0.99}Sb_{0.01}$, HfO_2, Ni_3Sn_4, Ni_3Sn_2, and $ZrHfO_2$ (from top to bottom). **(c)** 900°C sample with the markers assigned to $Zr_{0.75}Hf_{0.25}NiSn_{0.99}Sb_{0.01}$, HfO_2, Ni_3Sn_4, Ni_3Sn_2, and $ZrHfO_2$ (from top to bottom). **(d)** 950°C sample with the markers assigned to

$Zr_{0.75}Hf_{0.25}NiSn_{0.99}Sb_{0.01}$, HfO_2, Ni, $HfNi_2Sn$, Zr_2Ni_2Sn, and $ZrHfO_2$ (from top to bottom). **(e)** 1000°C sample with the markers assigned to $Zr_{0.75}Hf_{0.25}NiSn_{0.99}Sb_{0.01}$, HfO_2, Ni, $HfNi_2Sn$, Zr_2Ni_2Sn, and NiO (from top to bottom). **(f)** 1000°C sample with the markers assigned to $Zr_{0.75}Hf_{0.25}NiSn_{0.99}Sb_{0.01}$, HfO_2, Ni, $HfNi_2Sn$, Zr_2Ni_2Sn, and NiO (from top to bottom).

Table S2. Structural data from Rietveld refinements for $Zr_{0.75}Hf_{0.25}NiSn_{0.99}Sb_{0.01}$ samples: 800°C, 850°C, 900°C, 950°C, 1000°C and 1050°C, including lattice parameters (*a*), weight percent (*wt*%) for half-Heusler (HH) phases and oxides (i.e., HfO_2 and $ZrHfO_2$) phase, fractional occupancies (*Occ*) and isotropic thermal factor (B_{iso}/Å^2).

Sample		800°C	850°C	900°C	950°C	1000°C	1050°C
a (Å)		6.09953(5)	6.09713(4)	6.10128(4)	6.10360(4)	6.10209(4)	6.10348(4)
wt% (HH)		91.5	96.5	95.4	96.4	96.0	96.3
wt% (oxides)		2.9	1.2	2.6	2.4	2.5	2.6
Zr(4a)	*Occ*	0.7910	0.7840	0.7722	0.7837	0.7867	0.7723
	B_{iso}/Å^2	0.4(2)	0.3(3)	0.47(16)	0.45(22)	0.45(24)	0.36(15)
Hf(4a)	*Occ*	0.2090	0.2167	0.2277	0.2163	0.2133	0.2277
	B_{iso}/Å^2	0.4(2)	0.3(3)	0.47(16)	0.45(22)	0.45(24)	0.36(15)
Ni(4c)	*Occ*	1	1	1	1	1	1
	B_{iso}/Å^2	0.70(6)	0.48(5)	0.47(5)	0.58(6)	0.51(6)	0.64(5)
Ni(4d)	*Occ*	0	0.0045(72)	0.0146(62)	0.0213(74)	0.0182(74)	0.0225(60)
	B_{iso}/Å^2	0.70(6)	0.48(5)	0.47(5)	0.58(6)	0.51(6)	0.64(5)
Sn/Sb	*Occ*	1	1	1	1	1	1
(4b)	B_{iso}/Å^2	0.9(2)	0.6(3)	0.45(15)	0.53(21)	0.56(24)	0.84(17)
R_{Bragg} (%)		1.3	1.8	1.4	1.5	1.6	1.8
R_f (%)		1.1	1.5	1.4	1.4	1.3	1.4
χ^2		1.28	1.04	1.03	1.14	1.15	1.23

Table S3. The comparison of the lattice parameters with those reported values.[11–18]

Composition	Crystalline Form	Lattice Parameter (Å)	Sources
$Zr_{0.75}Hf_{0.25}NiSn_{0.99}Sb_{0.01}$	Polycrystalline	6.097 to 6.103	this work
ZrNiSn	Polycrystalline	6.113	PDF#23-1281, ref [11]
ZrNiSn	Polycrystalline	6.099	PDF#65-5987, ref [12]
ZrNiSn	Polycrystalline	6.106	Ref [13]
$Hf_{0.6}Zr_{0.4}NiSn_{0.98}Sb_{0.02}$	Polycrystalline	6.094	Ref [14]
$Hf_{0.25}Zr_{0.75}NiSn_{0.99}Sb_{0.01}$	Polycrystalline	6.098	Ref [15]
$ZrNi_{1.03}Sn_{0.99}Sb_{0.01}$	Polycrystalline	6.1	Ref [16]
ZrNiSn	Polycrystalline	6.109	Ref [17]
ZrNiSn	Single Crystal	6.1033	Ref [18]

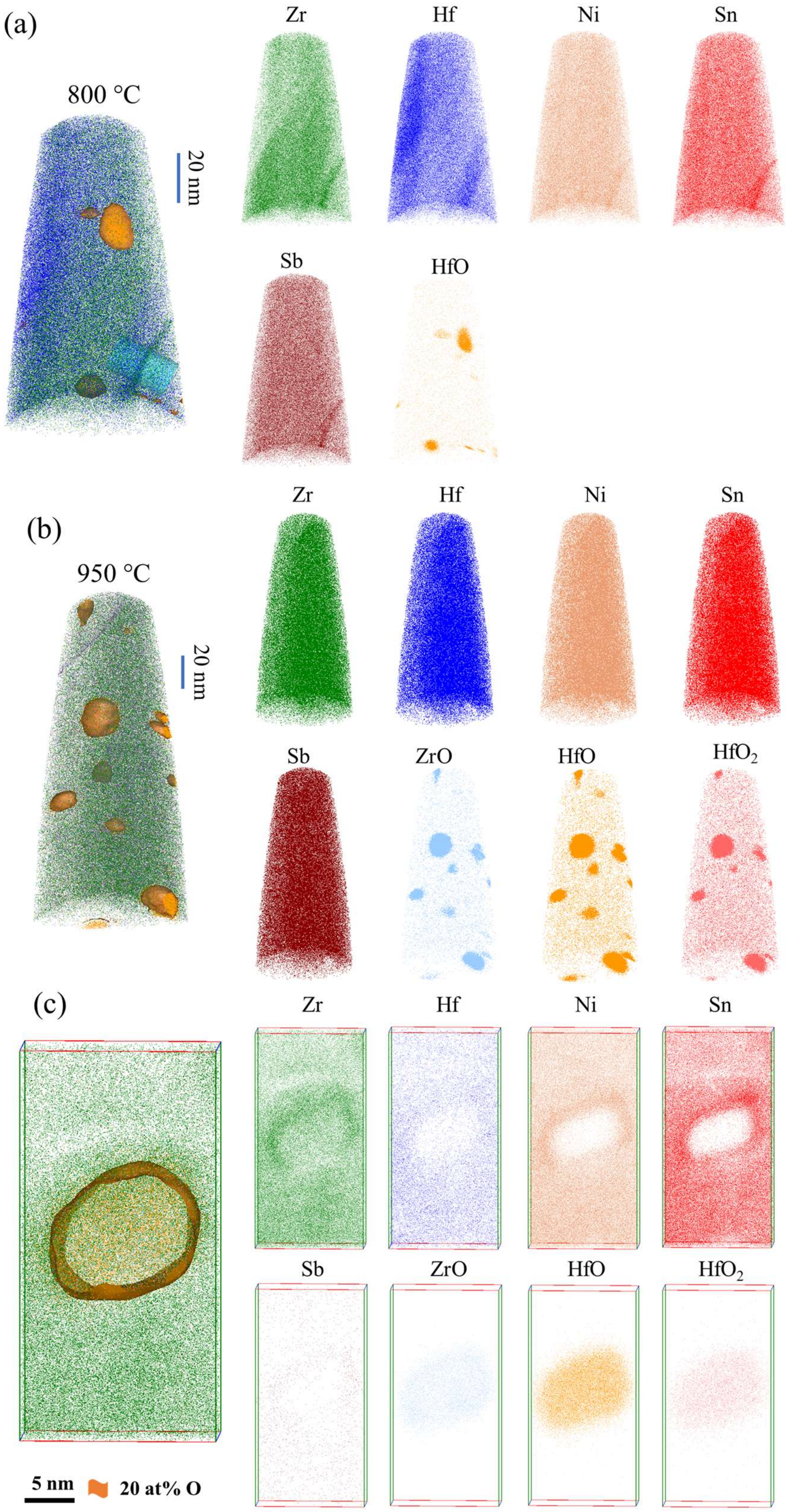

Figure S8. Atom probe tomography (APT) reconstruction and elemental distributions of Zr, Hf, Ni, Sn, Sb and oxides of Zr and Hf for $Zr_{0.75}Hf_{0.25}NiSn_{0.99}Sb_{0.01}$ samples: **(a)** 800°C, **(b)** 950°C, and **(c)** with a 10 nm-thick slice through the precipitates taken from (b).

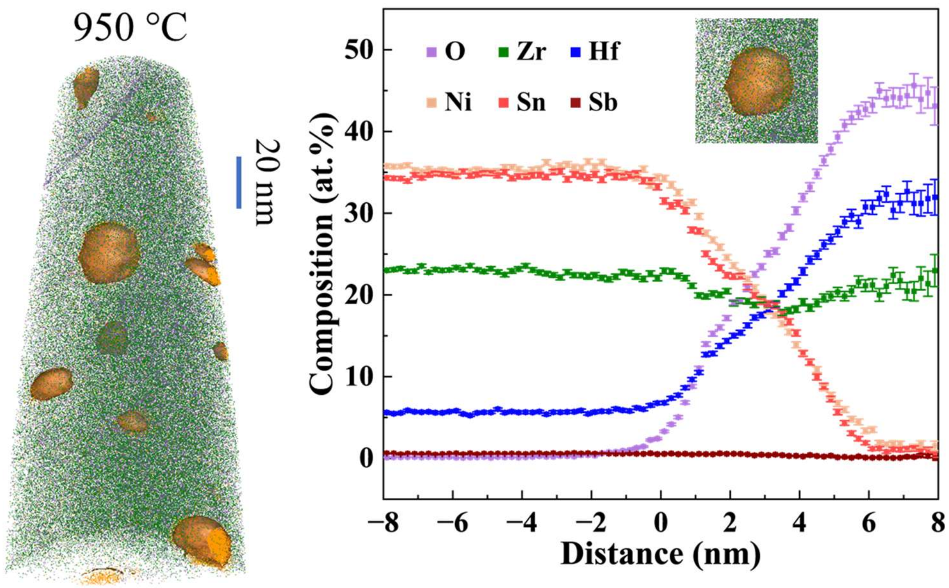

Figure S9. Atom probe tomography (APT) reconstruction and corresponding 1D concentration profiles across nanoprecipitates for $Zr_{0.75}Hf_{0.25}NiSn_{0.99}Sb_{0.01}$ samples: 950°C.

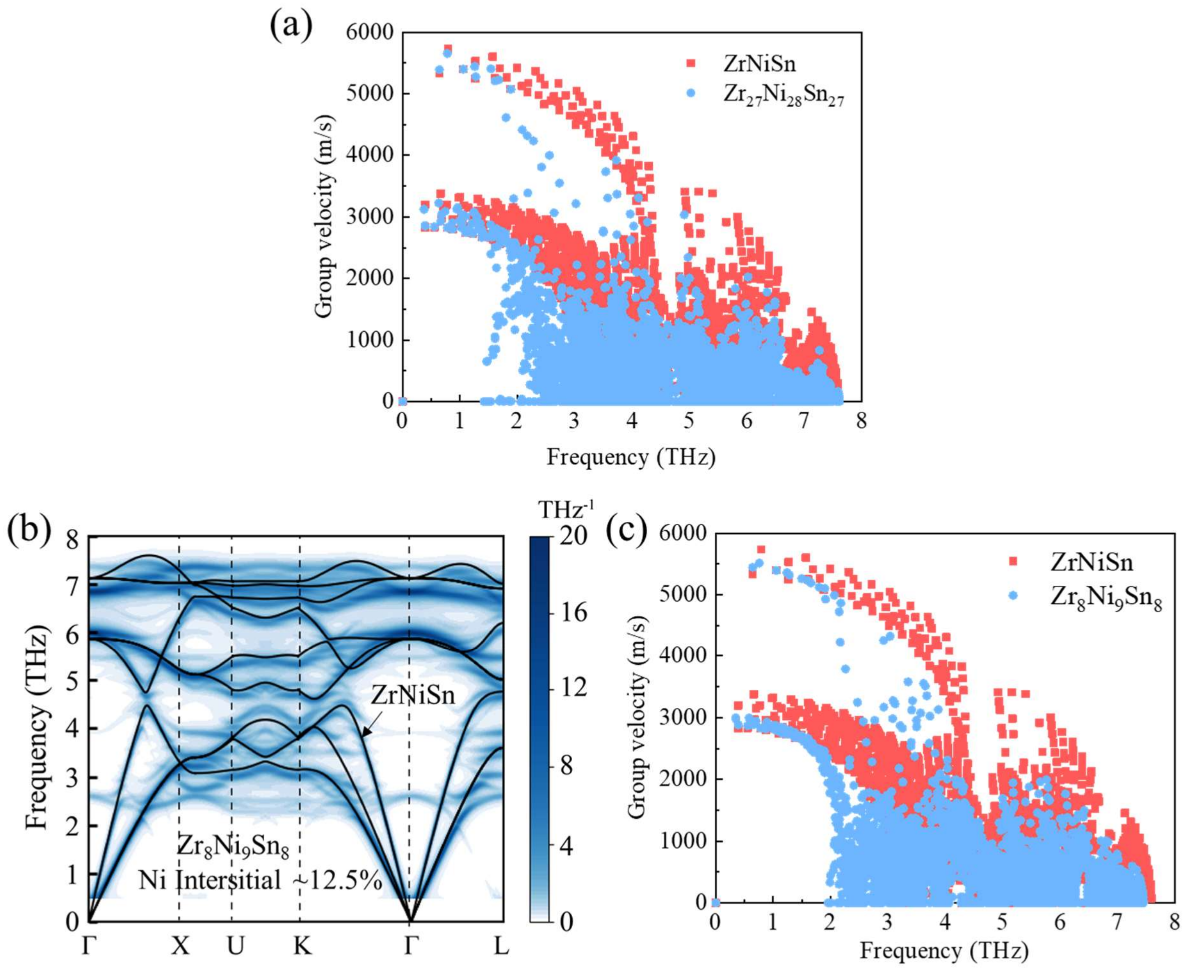

Figure S10. (a) Phonon group velocity of ZrNiSn and $Zr_{27}Ni_{28}Sn_{27}$ with a 4d Ni interstitial defect concentration of approximately 3.7%. **(b)** Phonon dispersion of ZrNiSn and $Zr_8Ni_9Sn_8$ with a 4d Ni interstitial defect concentration of approximately 12.5% and **(c)** corresponding phonon group velocity.

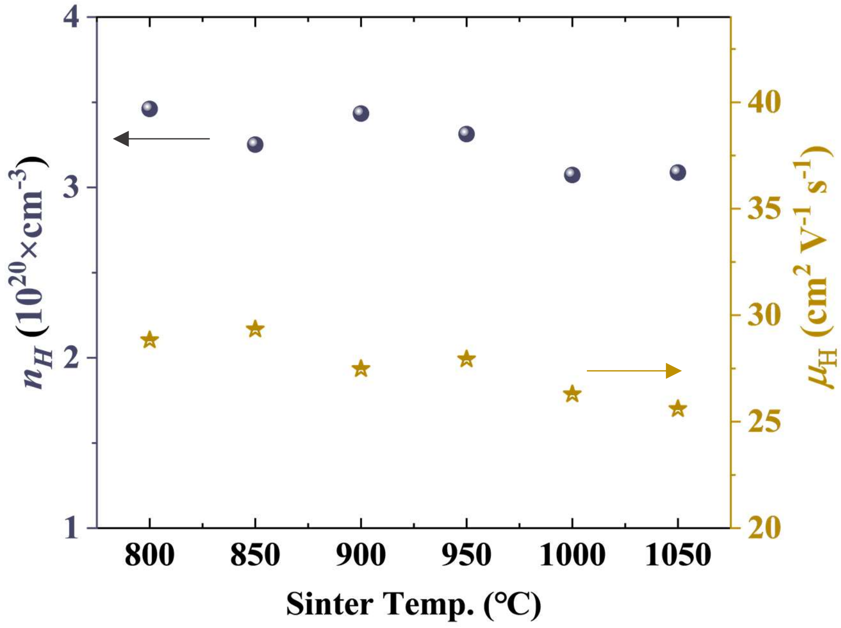

Figure S11. Room-temperature Hall carrier concentration (n_H) and Hall mobility (μ_H) of $Zr_{0.75}Hf_{0.25}NiSn_{0.99}Sb_{0.01}$ bulk samples: 800 °C, 850 °C, 900 °C, 950 °C, 1000 °C and 1050 °C.

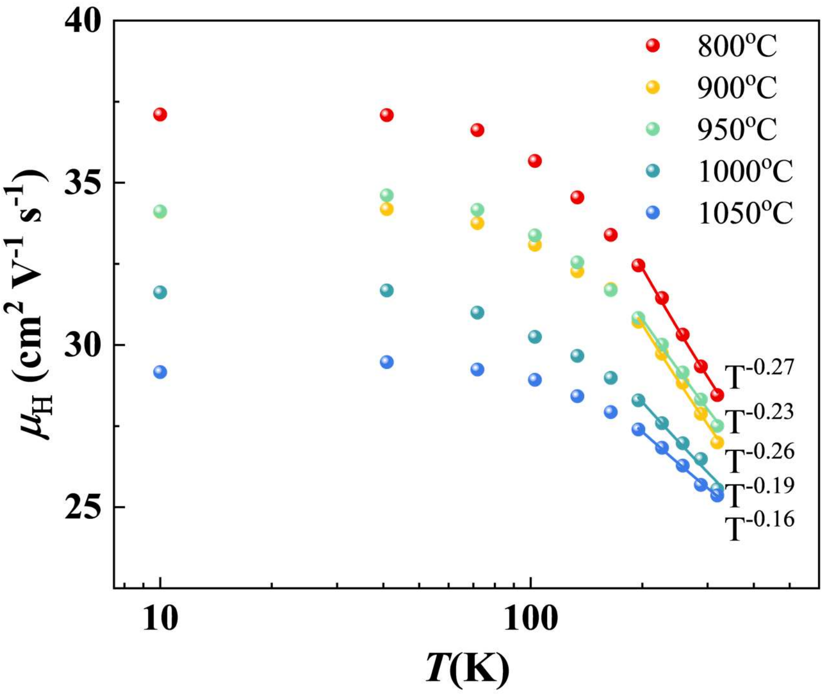

Figure S12. Temperature-dependent Hall mobility of $Zr_{0.75}Hf_{0.25}NiSn_{0.99}Sb_{0.01}$ bulk samples: 800 °C, 900 °C, 950 °C, 1000 °C and 1050 °C.

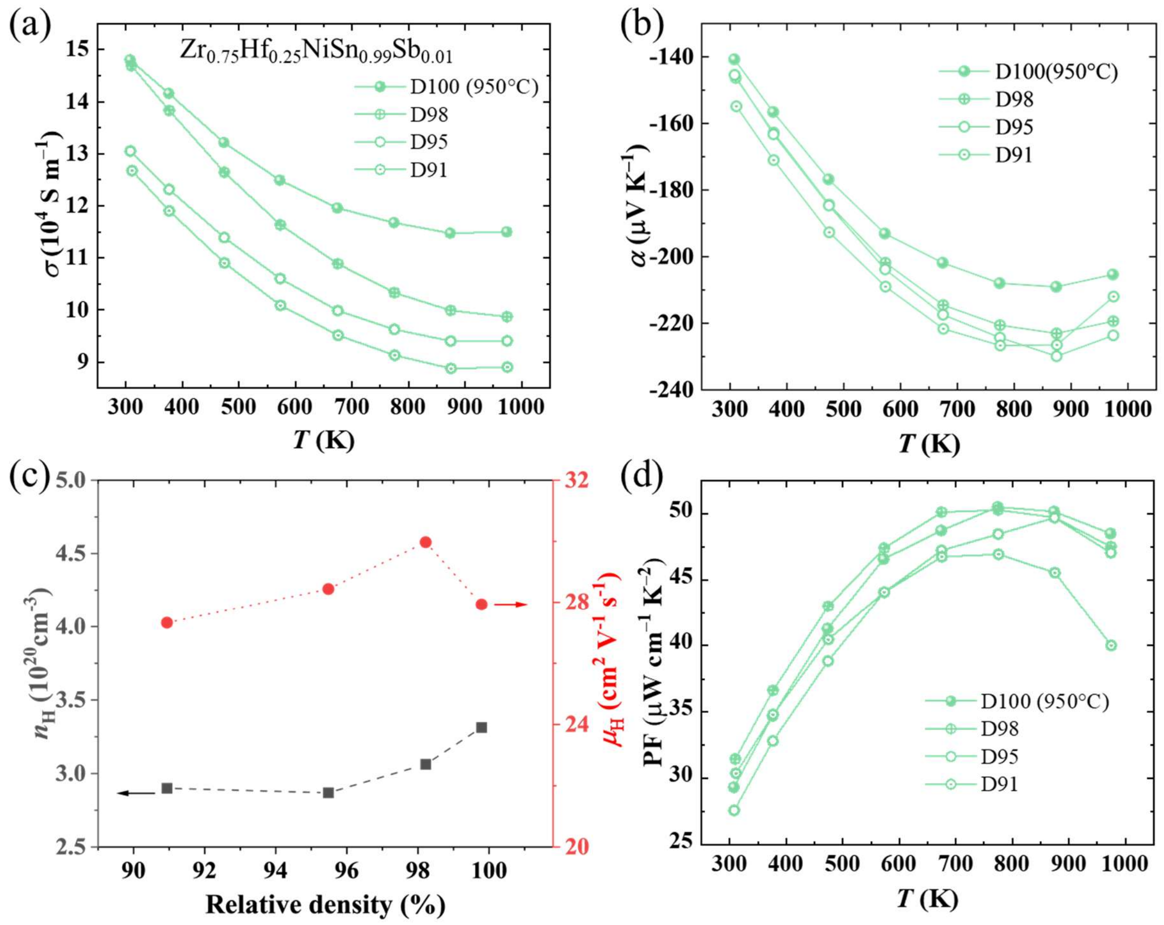

Figure S13. Thermoelectric performance of $Zr_{0.75}Hf_{0.25}NiSn_{0.99}Sb_{0.01}$ samples: D100, D98, D95, and D91. **(a)** Temperature-dependent electrical conductivity (σ); **(b)** Temperature-dependent Seebeck coefficient (α); **(c)** Room-temperature Hall carrier concentration (n_H) and Hall mobility (μ_H); **(d)** Temperature-dependent power factor (PF).

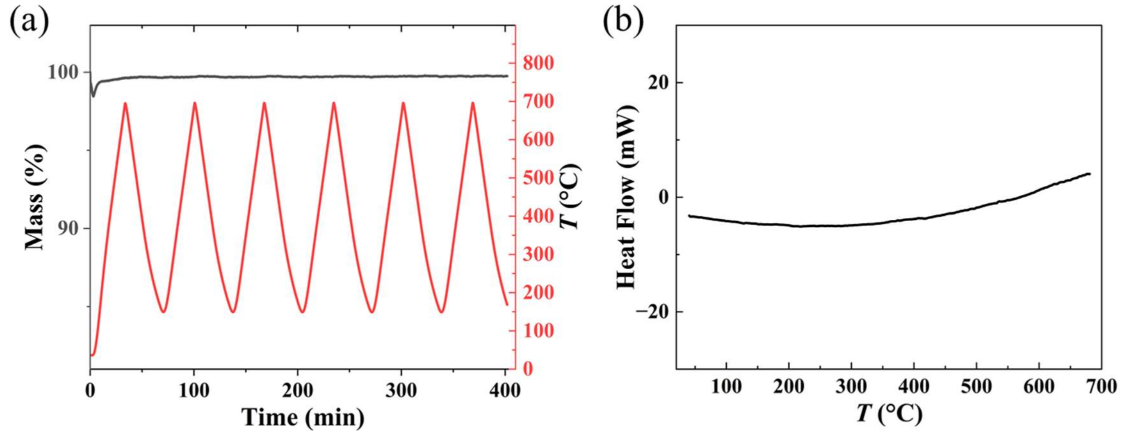

Figure S14 (a) TGA results of 6 heating–cooling cycles from 150 °C to 700 °C and **(b)** DSC curve from 35°C to 680 °C of sample D95.

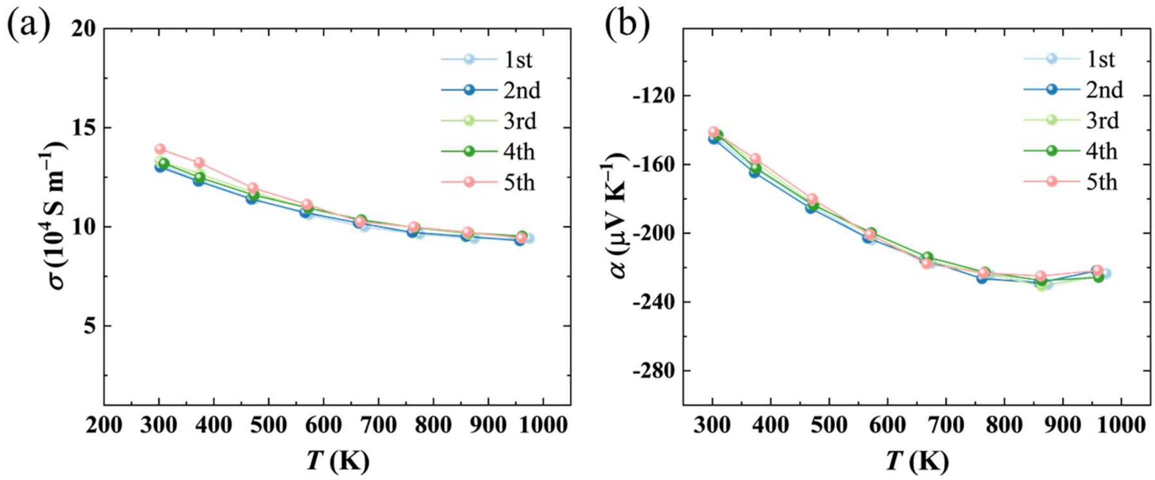

Figure S15 Repeated measurements of **(a)** electrical conductivity (σ) and **(b)** Seebeck coefficient (α) for porous sample (D95).

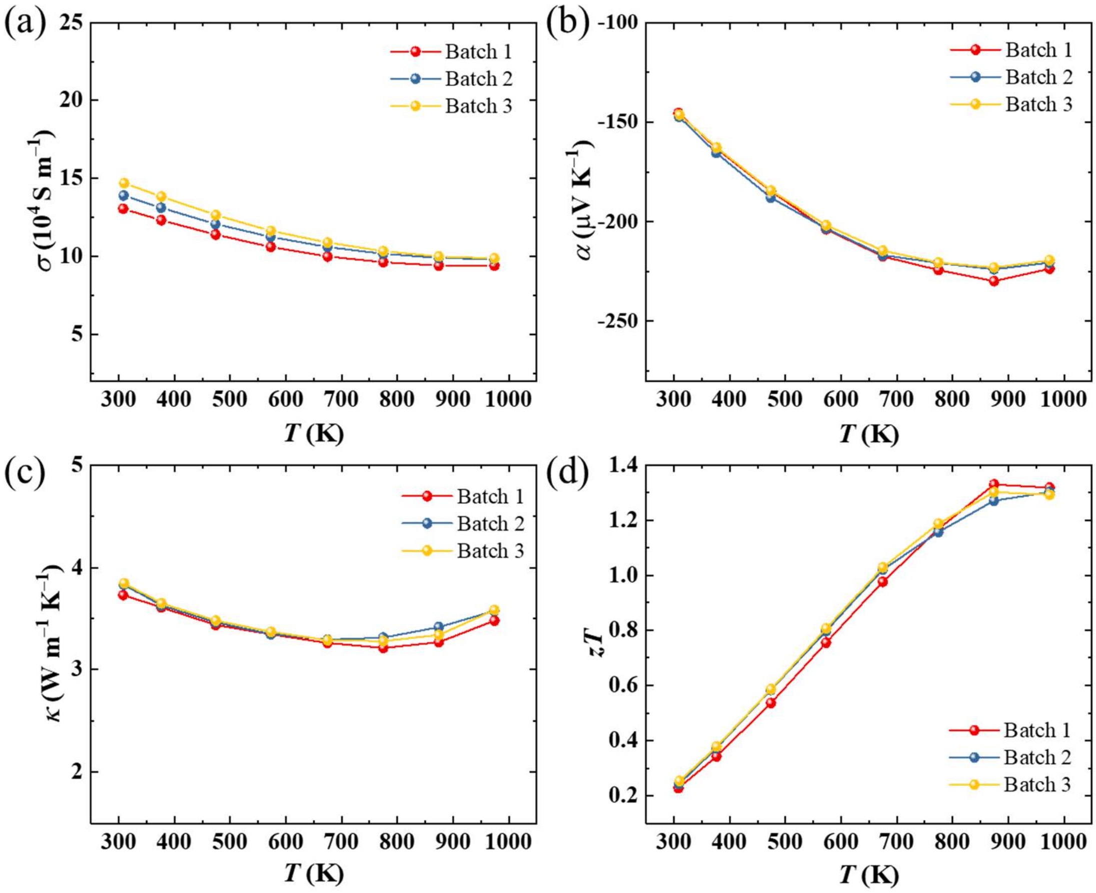

Figure S16 Thermoelectric performance of three batches of $Zr_{0.75}Hf_{0.25}NiSn_{0.99}Sb_{0.01}$ samples sintered at 950 °C with holding time 4 minutes (same as sample D95). **(a)** Temperature-dependent electrical conductivity (*σ*); **(b)** Temperature-dependent Seebeck coefficient (*α*); **(c)** Temperature-dependent thermal conductivity (*κ*); **(d)** Temperature-dependent dimensionless figure of merit (*zT*).

Table S3 Parameters in the Debye model for $Zr_{0.75}Hf_{0.25}NiSn_{0.99}Sb_{0.01}$.

Parameters	Descriptions	Unit	Values	Source (of information)
θ_D	Debye temperature	K	378	Calculated by Eq. 2−12
v_s	Average sound velocity	ms^{-1}	3379	exp.
d	Grain size	m	6×10^{-7}	estimated from SEM
d_p	Second phase size	m	6×10^{-7}	estimated from BSD
A	Point defect scattering parameter	/	0.11235	fitting
B	Umklapp scattering parameter	/	7.398	fitting
V_p	density of the nanoscale particles	m^{-3}	3.3×10^{22}	estimated from TEM and SEM
R	particle average radius	m	1×10^{-8}	estimated from TEM and SEM
C	nanoprecipitate scattering parameter	/	0.35	fitting
D	phase boundary scattering parameter	/	1.03	fitting

Table S4 Parameters in the Cahill model for $Zr_{0.75}Hf_{0.25}NiSn_{0.99}Sb_{0.01}$.

Parameters		
TA1 phonon velocity (m s^{-1})		3043.2
TA2 phonon velocity (m s^{-1})		3043.2
LA phonon velocity (m s^{-1})		5283.4
TA1 Debye temperature (K)		340.97
TA2 Debye temperature (K)		340.97
LA Debye temperature (K)		591.97
Minimum thermal	(300 K)	0.812
Conductivity (W m^{-1} K^{-1})	(973 K)	0.887

References

[1] H J Goldsmid, *Electronic refrigeration*, London : Pion, **1986**.
[2] H.-S. Kim, Z. M. Gibbs, Y. Tang, H. Wang, G. J. Snyder, *APL Materials* **2015**, *3*, 041506.
[3] Y. Li, T. Zhang, Y. Qin, T. Day, G. Jeffrey Snyder, X. Shi, L. Chen, *Journal of Applied Physics* **2014**, *116*, 203705.
[4] J. Callaway, *Phys. Rev.* **1959**, *113*, 1046.
[5] G. A. Slack, S. Galginaitis, *Phys. Rev.* **1964**, *133*, A253.
[6] G. S. Nolas, G. Fowler, J. Yang, *Journal of Applied Physics* **2006**, *100*, 043705.
[7] J. Yang, L. Xi, W. Qiu, L. Wu, X. Shi, L. Chen, J. Yang, W. Zhang, C. Uher, D. J. Singh, *npj Comput Mater* **2016**, *2*, 1.
[8] C. Fu, S. Bai, Y. Liu, Y. Tang, L. Chen, X. Zhao, T. Zhu, *Nat Commun* **2015**, *6*, 8144.
[9] N. Mingo, D. Hauser, N. P. Kobayashi, M. Plissonnier, A. Shakouri, *Nano Lett.* **2009**, *9*, 711.
[10] D. G. Cahill, S. K. Watson, R. O. Pohl, *Phys. Rev. B* **1992**, *46*, 6131.
[11] W. Jeitschko, *Metall Trans* **1970**, *1*, 3159.
[12] R. V. Skolozdra, Yu. V. Stadnik, E. Eh. Starodynova, *Ukrainskij Fizicheskij Zhurnal* **1986**, *31*, 1258.
[13] R. Yan, C. Shen, M. Widenmeyer, T. Luo, R. Winkler, E. Adabifiroozjaei, R. Xie, S. Yoon, E. Suard, L. Molina-Luna, H. Zhang, W. Xie, A. Weidenkaff, *Materials Today Physics* **2023**, *33*, 101049.
[14] T. J. Zhu, K. Xiao, C. Yu, J. J. Shen, S. H. Yang, A. J. Zhou, X. B. Zhao, J. He, *J. Appl. Phys.* **2010**, *108*, 6.
[15] S. Chen, K. C. Lukas, W. Liu, C. P. Opeil, G. Chen, Z. Ren, *Adv. Energy Mater.* **2013**, *3*, 1210.
[16] Y. Zhang, G. Peng, S. Li, H. Wu, K. Chen, J. Wang, Z. Zhao, T. Lyu, Y. Yu, C. Zhang, Y. Zhang, C. Ma, S. Guo, X. Ding, J. Sun, F. Liu, L. Hu, *Nat Commun* **2024**, *15*, 5978.
[17] X. Yang, Z. Jiang, J. Li, H. Kang, D. Liu, F. Yang, Z. Chen, E. Guo, X. Jiang, T. Wang, *Nano Energy* **2020**, *78*, 105372.
[18] C. Fu, M. Yao, X. Chen, L. Z. Maulana, X. Li, J. Yang, K. Imasato, F. Zhu, G. Li, G. Auffermann, U. Burkhardt, W. Schnelle, J. Zhou, T. Zhu, X. Zhao, M. Shi, M. Dressel, A. V. Pronin, G. J. Snyder, C. Felser, *Adv. Sci.* **2020**, *7*, 1902409.
